# Construction of circRNA-based ceRNA network to reveal the role of circRNAs in the progression and prognosis of metastatic clear cell renal cell carcinoma

**DOI:** 10.18632/aging.104107

**Published:** 2020-11-20

**Authors:** Xiyi Wei, Yuxiang Dong, Xinglin Chen, Xiaohan Ren, Guangyao Li, Yamin Wang, Yichun Wang, Tongtong Zhang, Shangqian Wang, Chao Qin, Ninghong Song

**Affiliations:** 1 The State Key Lab of Reproductive, Department of Urology, The First Affiliated Hospital of Nanjing Medical University, Nanjing 210029, China; 2 First Clinical Medical College of Nanjing Medical University, Nanjing 210029, China; 3 The Affiliated Kezhou People’s Hospital of Nanjing Medical University, Kezhou, Xinjiang 845350, China

**Keywords:** clear cell renal cell carcinoma, metastasis, ceRNA, CircRNAs, hsa_circ_0002286

## Abstract

CircRNAs are now under hot discussion as novel promising bio-markers for patients with clear cell renal cell carcinoma. The purpose of our study is to identify several circRNAs related to the metastasis and progression of clear cell renal cell carcinoma, and to further investigate the mechanism of their influence on tumor progression. The transcriptome data of ccRCC and clinical characteristics used in this study were downloaded from the The Cancer Genome Atlas and Gene Expression Omnibus database. A total of 114 circRNAs were found to be related to tumor initiation, progression and metastasis after the intersection. In addition, 14 miRNAs and 201 eligible mRNAs were selected as targets gene, respectively. CeRNA network was constructed based on 8 circRNAs, 14 miRNAs, and 201 mRNAs. Besides, another 6 hub genes were identified via the PPI network. It should be noted that only TRIM2 was confirmed as an independent prognostic factor, which was simultaneously significantly related to both clinical stage and pathological grade in clinical cohorts. Kyoto Encyclopedia of Genes and Genomes and Gene Ontology analysis indicated the possible function of TRIM2 in ccRCC progression, such as ubiquitin mediated protein hydrolysis, cell adhesion molecules, Th17 cell differentiation signaling pathway and so on. Gene set enrichment analysis analysis revealed that TRIM2 may be involved in ubiquitin mediated proteolysis, apoptosis, autophagy and citrate cycle TCA cycle. Hub circ_RNAs expressions were validated in ccRCC tissues and cell lines. Our study revealed that the hsa_circ_0002286 / has-mir-222-5p / TRIM2 axis played a critical role in the progression of ccRCC. Specifically, it may inhibit the metastasis and progression of ccRCC, which could serve as a potential therapeutic target.

## INTRODUCTION

Kidney cancer is one of the most prevalent malignant tumors in both men and women all over the world. Its incidence has been increasing in the past decade, comprising up to 2%-3% of all newly diagnosed tumor cases [1]. Histologically, clear cell renal cell carcinoma (ccRCC) is the prominent sub-type of kidney cancer, accounting for approximately 75% of kidney cancer cases [2]. More than 30% of patients with renal cancer exhibit metastasis at initial diagnosis [3]. High-grade renal cell carcinoma (RCC) represents a markedly high incidence of distant organ metastases [4]. The median survival time of patients with metastatic RCC is only 13 months, and the 5-year survival rate is less than 10% [5]. Although great development has been achieved in screening, diagnosis, surgery and various treatment, the clinical outcomes of advanced RCC remain poor [2, 6]. Therefore, in order to provide a better treatment for ccRCC patients, it is urgent to gain a deeper understanding of the mechanism of metastatic ccRCC.

Recently, the expansion of the biological understanding of non-coding RNA (ncRNA) has revealed its important roles in the process of tumorigenesis and progression [7, 8]. Circular RNAs (circRNAs) are a novel class of ncRNAs, which are mainly produced by the CIS deconvolution and cyclization of protein coding genes and other transcripts. While the new RNA is processing, the downstream splicing donor covalently connects with the upstream splicing receptor to generate cyclic ssRNA products [9]. In terms of function, although recent studies have reported some possible translation function of circRNA [10, 11], most of them are defined as untranslated ncRNA in cytoplasm [12, 13]. Few circRNAs have specific functions, and their contributions to physiology or pathology are just beginning to be elaborated [14–16]. Furthermore, circRNAs have not been extensively studied in the context of ccRCC biology or metastasis before.

Here, we described the expression and lineage of circRNA in primary and metastatic ccRCC, and investigated the mechanisms of circRNA to the development and metastasis of ccRCC.

## RESULTS

### Identification of differentially expressed CircRNAs (DECs)

**Figure 1:**
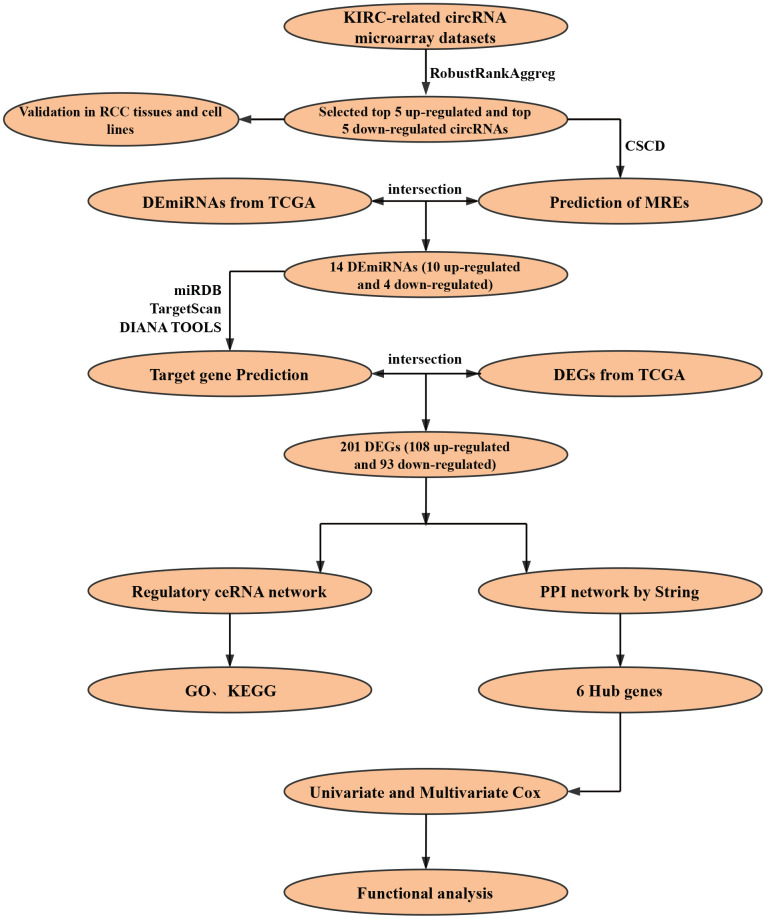
**Flow chart of research design.**

**Figure 2:**
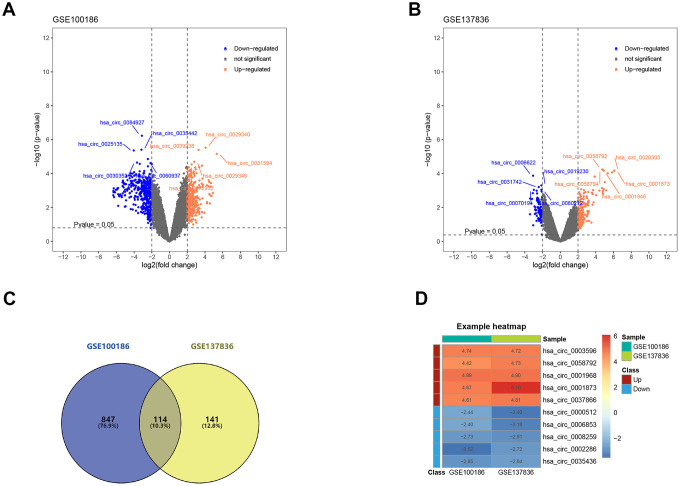
**Screening of differentially expressed circRNAs.** (**A**) The differential expression of circRNAs in ccRCC tissue samples compared with normal healthy kidney samples. (**B**) The differential expression of circRNAs in metastatic ccRCC tissue samples compared with local ccRCC tissue samples. (**C**) 114 circRNAs related to ccRCC progression and metastasis after the intersection. (**D**) Heatmap for top 5 up-regulated and top 5 down-regulated differentially expressed circRNAs. ccRCC: clear cell renal cell carcinoma.

The construction process of the whole ceRNA network is displayed in Figure 1. According to the defined criteria, a local ccRCC and a metastatic ccRCC microarray Gene Expression Omnibus (GEO) dataset (GSE100186 and GSE137836) were enrolled in the study (Table 1). After normalization of batch effect, in total, 961 circRNAs were differentially expressed between ccRCC and normal tissues in GSE100186 (Figure 2A), and 255 circRNAs were differentially expressed between metastatic ccRCC and primary tumor tissues in GSE137836 in total (Figure 2B). After the intersection, 114 CircRNAs were found to be related to tumor progression and metastasis (Figure 2C). The top 10 most differentially expressed circRNAs were selected for further analysis (Figure 2D) (Table 2).

**Table 1:** Essential characteristics of two circRNA microarray datasets from GEO.

**Data source**	**Platform**	**Author**	**Year**	**Region**	**Tissue information**	**No. of circRNAs**
GSE100186	GPL21825	Lv Q	2017	China	4 ccRCC tumor tissues VS 4 matched non-tumor tissues	13455
GSE137836	GPL21825	Li W	2019	China	3 metastatic ccRCC tumor tissues VS 3 primary ccRCC tumor tissues	12152

*ccRCC: clear cell renal cell carcinoma

**Table 2:** Basic information on the 10 differentially expressed circRNAs.

**CircRNA ID**	**Position**	**Genomic length**	**Strand**	**Best transcript**	**Gene symbol**	**Regulation**
hsa_circ_0003596	chr9: 137716445-137717750	369	+	NM_000093	COL5A1	Up
hsa_circ_0037866	chr16: 11778020-11830089	1980	-	NM_015914	TXNDC11	Up
hsa_circ_0058794	chr2: 236626200-236659132	451	+	NM_001037131	AGAP1	Up
hsa_circ_0001968	chr11: 68359043-68367962	407	+	NM_001164160	PPP6R3	Up
hsa_circ_0001873	chr9: 93637042-93639999	2957	-	NM_001174167	SYK	Up
hsa_circ_0008259	chr13: 76287317-76335174	248	+	NM_005358	LMO7	Down
hsa_circ_0002286	chr1: 107866903-107867544	641	+	NM_001113226	NTNG1	Down
hsa_circ_0035436	chr15: 57753877-57754090	213	+	NM_001252335	CGNL1	Down
hsa_circ_0006853	chr14: 20811282-20811360	78	-	NR_002312	RPPH1	Down
hsa_circ_0000512	chr14: 20811282-20811436	154	-	NR_002312	RPPH1	Down

### Identification of target miRNA and target mRNA

**Figure 3:**
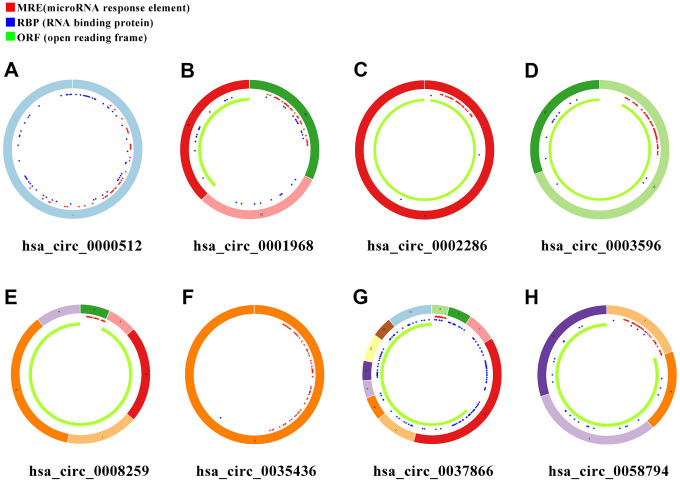
**Schema graphs of eight circRNAs downloaded from Cancer-Specific CircRNA database (CSCD).** (**A**) hsa_circ_0000512. (**B**) hsa_circ_0001968. (**C**) hsa_circ_0002286. (**D**) hsa_circ_0003596. (**E**) hsa_circ_0008259. (**F**) hsa_circ_0035436. (**G**) hsa_circ_0037866. (**H**) hsa_circ_0058794. Red spots represent miRNA response elements (MRE), blue spots represent RNA binding protein (RBP), and green curves represent open reading frame (ORF).

**Figure 4:**
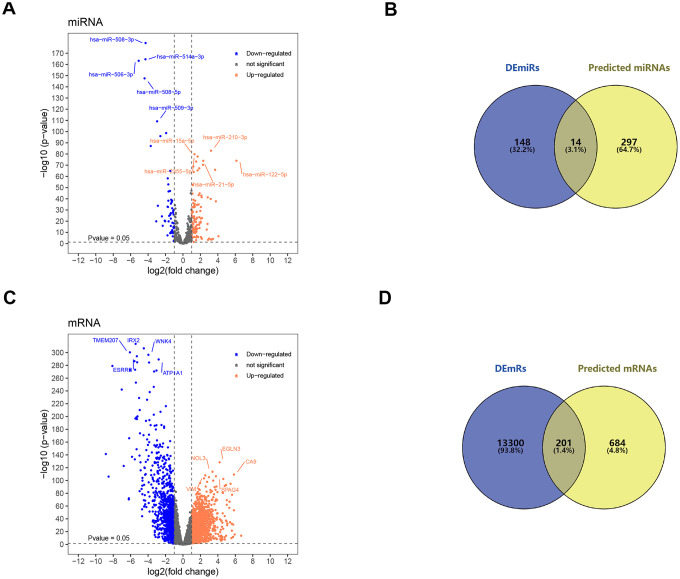
**Screening of target miRNAs and target mRNAs.** (**A**) Volcano map for 162 differentially expressed miRNA with 105 upregulated miRNAs and 57 downregulated miRNAs. (**B**) Intersection of predicted miRNA and differential miRNA. (**C**) Volcano map for 13501 differentially expressed mRNAs with 10191 up-regulated mRNAs and 3310 down-regulated mRNAs. (**D**) Intersection of predicted mRNA and differential mRNA. DEmiRs: differentially expressed miRNA; DEmRs: differentially expressed mRNAs.

In order to search for microRNA response element (MRE) and find its target miRNAs, 10 selected circRNAs were predicted via Cancer-Specific CircRNA database (CSCD), through which only eight can be identified. The pattern map of eight DECs containing MRE, RNA binding protein and open reading frame information was downloaded from CSCD (Figure 3). Based on The Cancer Genome Atlas (TCGA) kidney renal clear cell carcinoma (KIRC) database, quantitative data of miRNA and mRNA expression in 537 ccRCC samples were obtained by sequencing (miRNA-seq and mRNA-seq). R software was utilized to screen differentially expressed miRNAs (DEmiRs) and differentially expressed mRNAs (DEmRs) in ccRCC patients extracted from TCGA. After intersection of predicted miRNA and differential miRNA, 14 miRNAs were selected as target miRNAs (Figure 4A, 4B, Supplementary Figure 1). Then, MiRDB, TargetScan and DIANA databases were utilized to predict the target mRNA of these 14 miRNAs respectively. Finally, 201 eligible target mRNAs were identified as the final target genes by crossing with differential mRNA (Figure 4C, 4D) (Table 3).

**Table 3:** Target genes obtained from the intersection of prediction and differential genes.

**Up-regulated DEGs**	MDM4,CSF1,CX3CL1,CELF2,VAMP1,PLEKH02, TET3,TFPI,C5orf64,CHRNA5,CXCR1,PSTPIP2,MEX3B,SLC41A2,FAM198B, SERPINE2,GIPC3,SLEC1A,PLA2G6,FMNL3,MGAT3,GRM2, MEF2C,OAS2,SPIB,DUSP10,XIRP1,RNF165,AATK,ZNF177, TCHH,CHST1,CD28,RUNX2,DNM1,AFF3,HECW2, COR07,FSCN1,PR0CR,BCL11B,ANKRD23,PDE1B,JPH3,RGS18, DGKI,MPPED1,ERVW-1,LV6E,CD200,INPP5D,FSTL3, RASSF5,LRRC3B,KLF17,ELM01,GBP1,KCNJ2,PDGFD,C7orf61, PDK1,POU2F2,ITGA5,ARL11,TTYH3,FBX041,C18orf63,CDCA7,XAF1, PKP1,E2F7,MSR1,RELT,ADAM11,ASF1B,E2F1,KIF21B, HSPB8,VASH1,HLA-D0B,LRAT,THBS4,,EDA2R,NR5A1, CD6.CPLX2,DSG3,SLC12A5,LZTS1,HOXA13,DLL4,OSCAR, RGS5,C20orf197,CD84,GABRE,DMBX1, PITX1,TRPA1,DCLK3,ATP2B2,LOX,C6orf223, GBP5,RBM46,DMRT1,GRIK3,OPN4
**Down-regulated DEGs**	CALB1,CLDN19,FLRT1,GRHL2,CNTN1,EYA4, HOXB9,PAK6,KCNK10,TMEM178A,FOXJ1,ESRRG,FGF9, CHRNA4,SCN2A,SIM1,DLK1,MPP7,NKD1,OXGR1,GABRA4,GRIK2, AQP5,PLCL1,PLXNA4,LINGO2,COBLL1,CACNA2D2,AQP3,HOXB6,SHISA3, PCDHA12,HOXB5,RAB11FIP4,PPM1H,XK,SAMD12,C5orf47, TRIM2,MFAP3L,PPP1R1C,FAXC,CHODL,PLAG1,KLHL3,PALM,CACNB4, RHOBTB3,GATA2,IGDCC3,RERGL,TRDN,KL, FCAMR,C14orf132,SBK1,MAN1A1,CADM1,L2HGDH, CPEB3,GABRB2,CNP,KIF26B,RIMS4,USP46,ZDHHC15, SGPP1,PTPN3,SNRPN,PTPN4,LMO7,C5orf52, SLIT2,PTGS2,KCNA1,PDHA1,NDUFS1,DLG2, TMEM56,DOCK3,GPT2,FLRT2,PCDHA11,CLMP, HEPACAM2,MOB1B,DENND1B,SCUBE3,MAPK10,TOM1L2, PRKX,CHD3,ZDHHC3

### Functional annotation of the target miRNA and target mRNA

**Figure 5:**
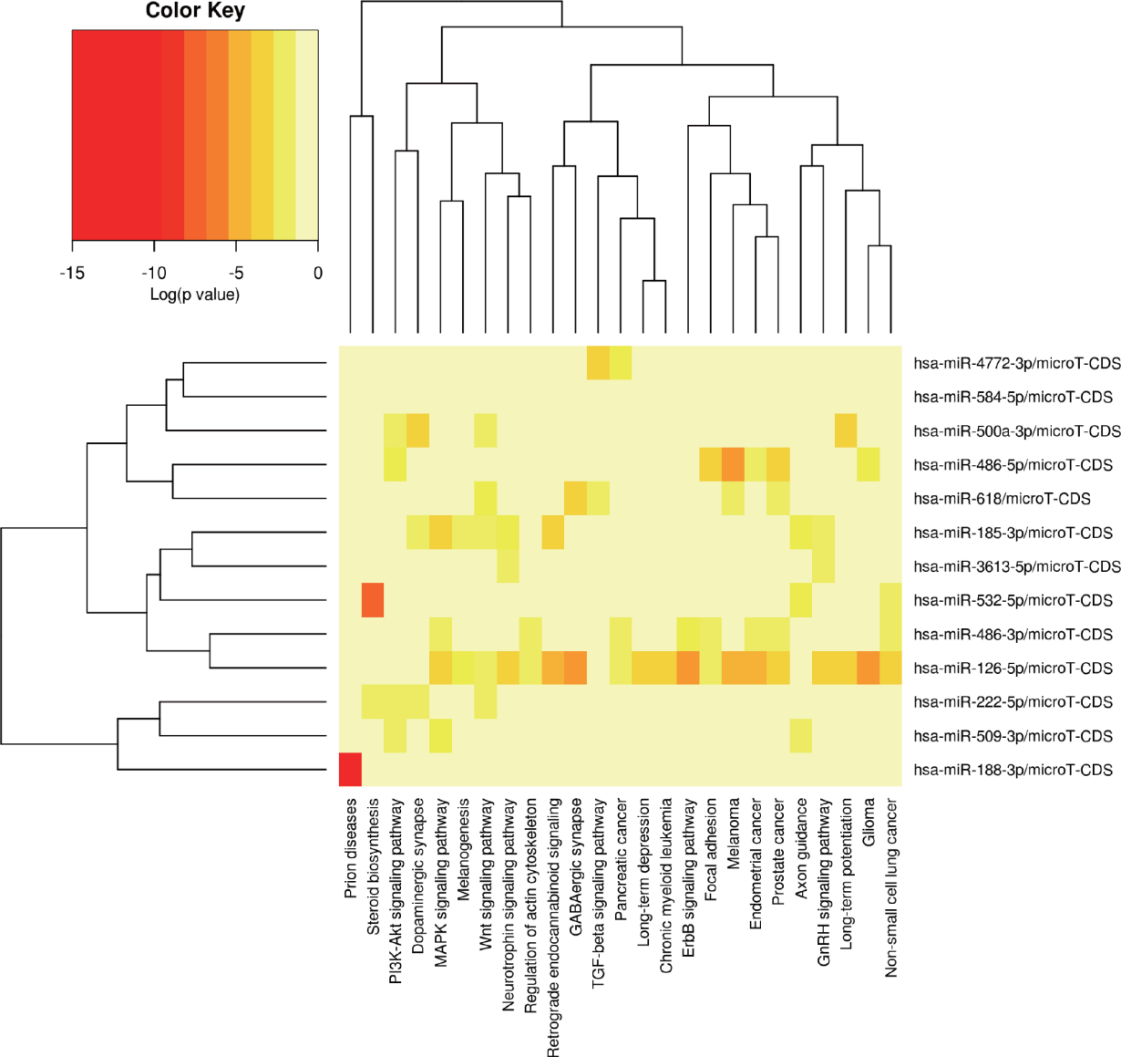
**The potential signal pathways 13 miRNAs may be involved in predicted via miRPath.**

MiRPath was applied to explore the possible signal pathways in which the 14 miRNAs may be involved. The results showed that several miRNAs were closely associated with tumor related pathways (Figure 5). In order to fully understand the biological relevance of these 201 target genes, we conducted Kyoto Encyclopedia of Genes and Genomes (KEGG) and Gene Ontology (GO) analysis. Based on the results of DAVID, the top enriched biological process (BP), cellular components (CC) and molecular function (MF) terms were: regulation of synaptic plasticity, integral component of plasma membrane and fibroblast growth factor receptor binding (Supplementary Figure 2A, 2B). The principal enriched biological pathways were Nicotine addiction (Supplementary Figure 2B).

### Construction of ceRNA network and PPI network

**Figure 6:**
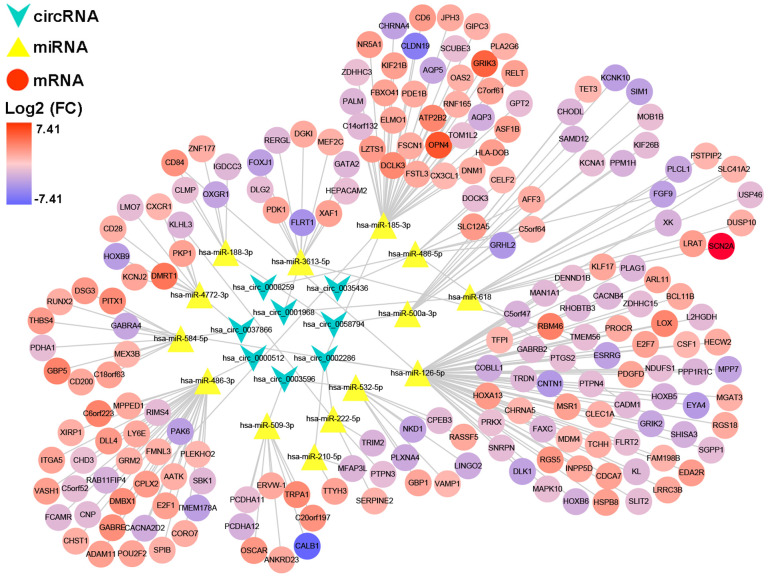
**A circRNA/miRNA/mRNA regulatory network.** The network consists of eight circRNAs, 14 miRNAs, and 201 mRNAs. CircRNAs, miRNAs, and mRNAs are respectively represented by rounded rectangles, diamonds, and ellipses. Their volumes and colors are determined by fold changes.

**Figure 7:**
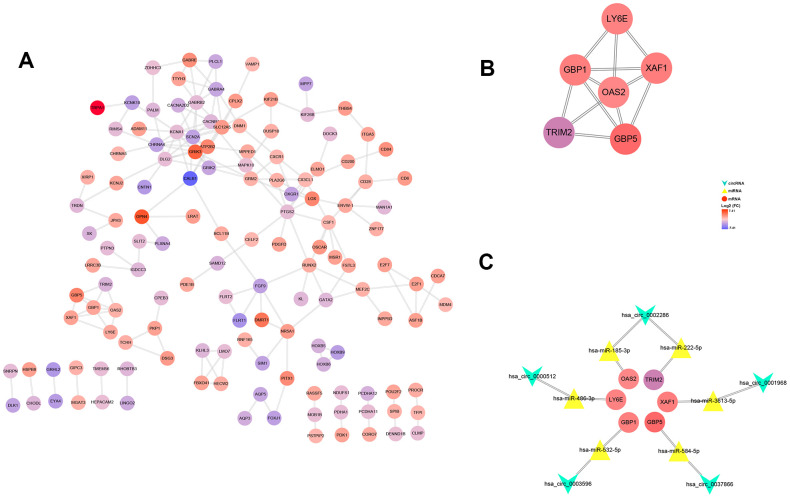
**PPI network, hub-genes, and circRNA/miRNA/hub-genes axes.** (**A**) The PPI network of 201 genes. (**B**) Six hub-genes (COL10A1, COL5A2, COL4A1, and COL3A1) in the PPI network. (**C**) The circRNA / miRNA / hub-genes axes.

After multiple filters, 8 circRNAs, 14 miRNAs and 201 target genes were screened out for further study. Then, a ceRNA: circRNA / miRNA / mRNA network was established by combining the circRNA / miRNA interaction and miRNA/mRNA interaction, which initially showed the overall view of regulatory network (Figure 6). String database provided a visual PPI regulatory network of target genes. We visualized the PPI network results via the Cytoscape software (Figure 7A). According to app MCODE, the six central genes that played crucial roles in the network were TRIM2, XAF1, GBP1, GBP5, LY6E and OAS2 (Figure 7B, Figure 7C).

### Cox regression analysis and clinical correlation analysis

**Figure 8:**
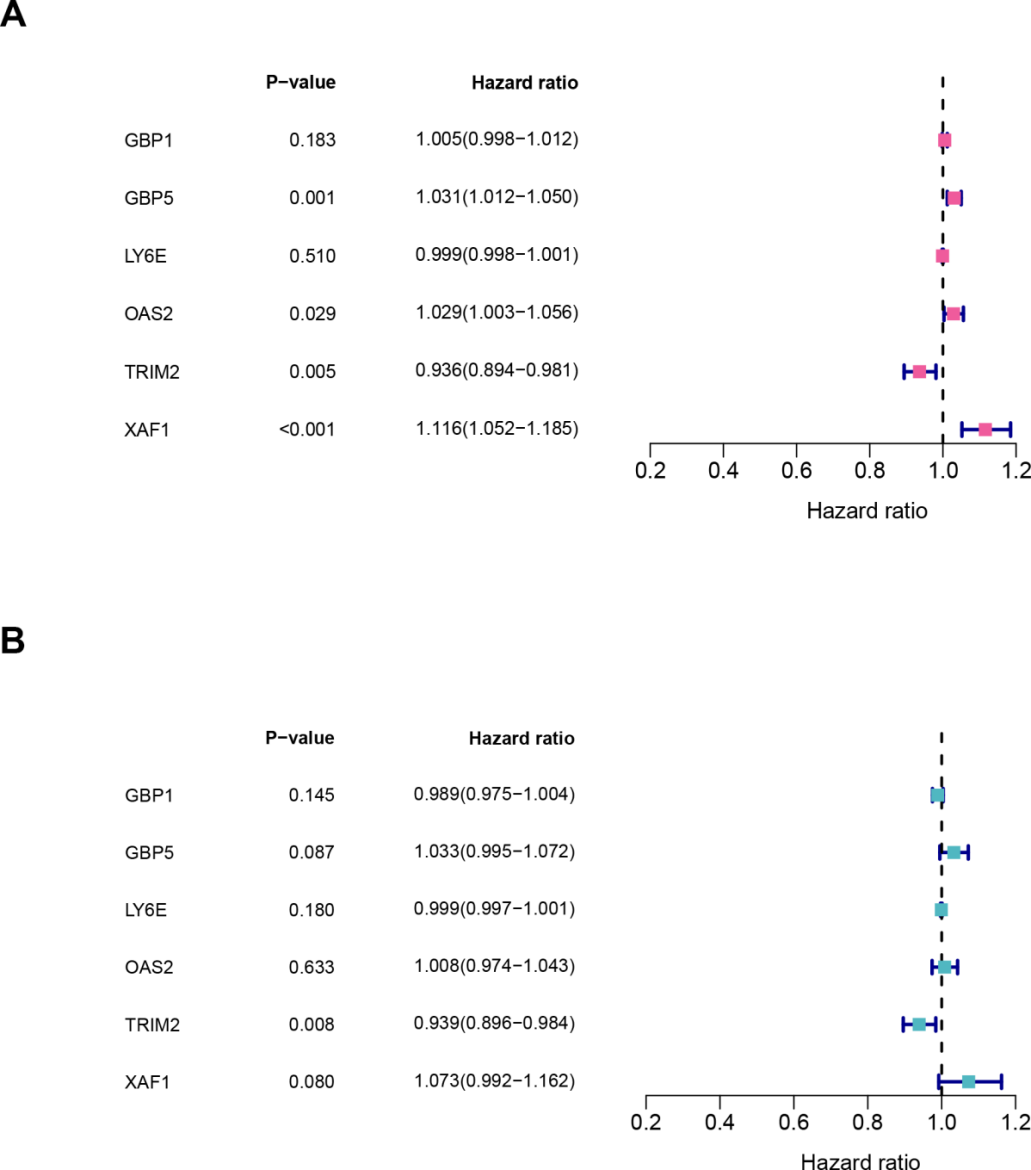
**Characteristics of six hub genes in the prognostic risk models.** (**A**) univariate cox and (**B**) multivariate cox regression coefficients and hazard ratios of the six hub genes.

**Figure 9:**
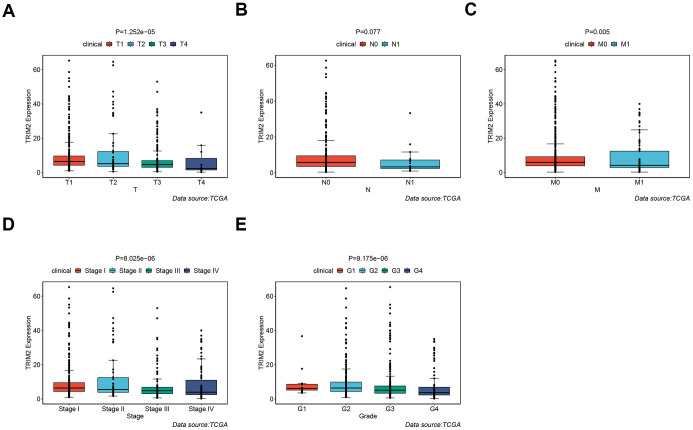
**The different expression of TRIM2 in clinical TNM stage, pathological stage and grade of patients with TCGA KIRC.** (**A**) T stage. (**B**) N stage. (**C**) M stage. (**D**) Pathological stage. (**E**) Pathological grade.

It should be noted that the expression level of all six hub-genes were up-regulated in ccRCC tissues, except TRIM2 (Supplementary Figure 3). Among them, only TRIM2 and XAF1 were significantly related to overall survival (OS). Specifically, high expression of TRIM2 predicted better survival, while XAF1 is negatively correlated with survival (Supplementary Figure 3B, 3D). Cox proportional hazards model was then applied to screen hub genes independent of survival. According to the model, only TRIM2 was confirmed as independent prognostic factors for survival (Figure 8). Thus, TRIM2 was identified as the star hub gene in our research. The association between each subset of TNM and stage and grade and TRIM2 was then analyzed by R software via the Wilcox test. We found that the expression level of TRIM2 significantly stepwise decreased in each subgroup of TNM, stage and grade (Figure 9). In the tumor stage, metastasis stage, pathological stage and grade, we found that TRIM2 decreased significantly (all p values < 0.05) (Figure 9A, 9C–9E). Although TIRM2 gradually decreased in the node stage subgroup but there was no statistical difference (P=0.074, Figure 9B).

### GO and KEGG analysis of TRIM2 co-expressed genes and gene set enrichment analysis (GSEA)

**Figure 10:**
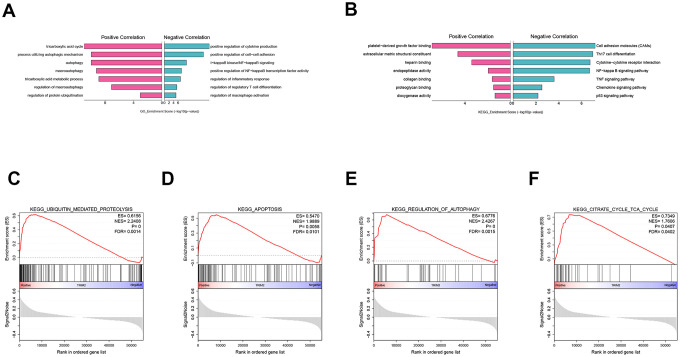
**GO, KEGG analysis of TRIM2 co-expressed genes and GSEA analysis.** (**A**) GO. (**B**) KEGG. (**C**) KEGG_UBIQUITIN_MEDIATED_PROTEOLYSIS. (**D**) KEGG_APOPTOSIS. (**E**) KEGG_REGULATION_OF_AUTOPHAGY. (**F**) KEGG_CITRATE_CYCLE_TCA_CYCLE. GO: gene ontology; KEGG: kyoto encyclopedia of genes and genomes; GSEA: Gene set enrichment analysis; ES: Enrichment score; NES: Normalized enrichment score; FDR: False discovery rate.

To explore the biological function of TRIM2 in progression and metastasis of ccRCC, we performed a GO and KEGG analysis of TRIM2 co-expressed genes based on the TCGA KIRC cohort, as well as GSEA analysis. GO analysis indicated that on one hand, TRIM2 can positively regulate the cycle of tricarboxylic acid cycle, autophagy and protein ubiquitination (Figure 10A). On the other hand, it may also negatively regulate the production of cytokines, cell-cells adhesion and NF-kappaB signaling. With regard to KEGG pathway, we found that TRIM2 can negatively regulate cell adhesion molecules, Th17 cell differentiation and Cytokine−cytokine receptor interaction (Figure 10B). Furthermore, the most cardinal pathway identified by GSEA was ubiquitin mediated proteolysis, apoptosis, autophage and tricarboxylic acid cycle (Figure 10C–10F) (Supplementary Figure 4).

### Relationship between TRIM2 and drug sensitivity

The Genomics of Drug Sensitivity in Cancer (GDSC) database was exploited to analyze the relationship between the drug sensitivity of ccRCC cell lines and the expression of TRIM2. The results revealed that the high expression of TRIM2 increased not only the resistance of ccRCC cell lines to paclitaxel, S-Trityl-L-cysteine, pyrimethamine, GW843682X and bortezomib (P < 0.05), but also the sensitivity of ccRCC cell lines to salubrinal, GNF-2, XMD8-85, PHA-665752 and BEZ235 (Supplementary Figure 5).

### Validation of circRNAs and TRIM2 in tissues and cell lines

**Figure 11:**
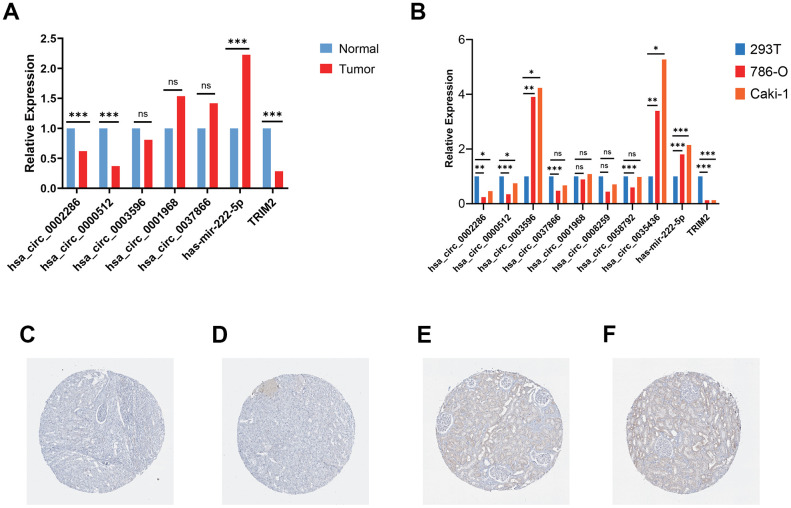
**Validation of circRNAs and TRIM2 in ccRCC tissues and cell lines.** (**A**) Bar plot for the relative expression of five hub circ_RNAs in ccRCC tissues and normal tissues. (**B**) Bar plot for the relative expression of 8 circ_RNAs in ccRCC cell lines and normal 293T cell line. (**C**, **D**) Immunohistochemical analysis of TRIM2 in ccRCC tissues. (**E**, **F**) Immunohistochemical analysis of TRIM2 in normal kidney tissues.

RT-qPCR was carried out in 15 pairs of ccRCC tissues and normal kidney tissues and 3 cell lines, including 2 tumor cell lines and 1 normal renal cell line. In tumor tissues, the relative mRNA expression levels of hsa_circ_0002286, hsa_circ_0000512 and TRIM2 were lower than those in normal tissues (P < 0.01), while the expression levels of hsa-mir-222-5p was significantly increased in tumor tissues (P < 0.01) (Figure 11A). Additionally, the relative mRNA expression levels of hsa_circ_0002286, hsa_circ_0000512, hsa_circ_0003596, hsa_circ_0035436, hsa-mir-222-5p and TRIM2 were markedly different between tumor cell lines and 293T cell line (P <0.05) (Figure 11B). It was consistent with the results predicted by bio-informatics methods. Moreover, The Human Protein Atlas (HPA) database distinctly revealed that in ccRCC tissues (Figure 11A and 11B), the expression levels of TRIM2 were significantly lower than that in the normal kidney tissue (Figure 11C and 11D).

## DISCUSSION

ccRCC is a heterogeneous disease with different ethnic characteristics, which originate from renal tubule epithelial cells [17]. It is estimated that ccRCC accounts for the majority of RCC related deaths [18]. Although radical nephrectomy has been proved to be a definitive treatment for localized RCC, many patients may still experience progress or recurrence after surgical operation. Given that targeted treatment for advanced and metastatic ccRCC has been fully developed, the response to treatment is diverse [19]. It is universally known that the determination of molecular mechanism and applicable prognostic factors may be the crucial lynchpin for the treatment of ccRCC [20]. It has been extensively documented that cancer is a gene disease. CircRNAs are important regulators of gene expression during genetic flow processing in living cells and interact with major cellular pathways (e.g. proliferation, differentiation, apoptosis). Consequently, they may take part in the development of many human malignancies. Understanding of the roles of circRNAs in ccRCC remains a fundamental unmet medical need. To this end, we screened the circRNAs relevant to the progression and metastasis of ccRCC and a ceRNA regulation network was conducted.

Based on the published databases (GEO and TCGA), we identified the differentially expressed circRNAs, miRNAs and mRNAs in ccRCC. Through the integrated analysis of multiple databases, a ccRCC centered ceRNA network (circRNA / miRNA / mRNA) was established. We also established a PPI network to highlight the hub genes that may pertain to the metastasis of ccRCC. In addition, a functional analysis was conducted to speculate the biological function of hub genes in ccRCC. Ultimately, a core tumor suppressor axis (circRNA_0002286 / miR-222-5p / TRIM2) was identified. Our research provided new notions into the role of circRNAs-related ceRNA networks in ccRCC and identify potential therapeutic targets.

Several lines of evidence have revealed that circRNAs occur disorders in various cancers, including ccRCC, which may be related to the tumor progression and dissemination. For instance, Xue et al. reported the role of circRNA-AKT3 in ccRCC. They confirmed that circ-AKT3 enhanced E-cadherin expression through competitive binding of mir-296-3p, thus inhibiting the metastasis of ccRCC [21]. Among the eight circRNAs we selected, only circRNA_0008259 has been studied. Zhang et al. estimated the expression spectrum of circRNAs and its role in keloid dermal fibroblasts [22]. Hsa_circRNA_0008259 were verified to be significantly reduced in keloid dermal fibroblasts. Their results suggest that circRNAs may regulate the occurrence and development of keloids through apoptosis, focal adhesion, PI3K-Akt and metabolic pathways, which have been extensively documented in tumor progression and metastasis. However, other circRNAs found in our ceRNA network have not been explored yet. Therefore, the regulatory mechanism of the rest circRNAs in ccRCC needs further investigation in the future.

MiRNAs, a kind of endogenous non coding RNA, affect the occurrence and development of various cancers by regulating the expression level of oncogenes and tumor suppressor genes [23]. Kong et al. identified LncRNA ATP2B1 / miR-222-5p / TAB2 and LncRNA HUWE1 / miR-222-5p / TAB2 as potential ceRNA networks related to NSCLC resistance [24]. Ji et al. found that FGFR4 and EGFR were targets of mir-486-3p, and increased expression of mir-486-3p can induce apoptosis of hepatoma cells [25]. Qu et al. indicated that Linc00152 can be used as an endogenous RNA competing with mir-185-3p to increase the expression of fascial-actin-binding-protein 1 (FSCN1), so as to promote the malignant proliferation and metastasis of colorectal cancer cells [26]. Zhai et al. found that miR-532-5p suppressed RCC cell proliferation by impeding the ETS1-mediated KRAS-NAP1L1/P-ERK axis [27]. Wei et al. demonstrated that miR-584-5p functioned by targeting KCNE2 directly, and mediates the proliferation, migration and invasion of hepatocellular carcinoma (HCC) cells [28]. The remaining miRNAs have also been found to be related to tumorigenesis and tumor progression [29].

A PPI network was developed to investigate the interaction between the target mRNA we identified, and the six hub genes with the highest connectivity were selected. It has been reported that some of these genes are closely related to RCC, such as TRIM2 and XAF1. Xiao et al. demonstrated that exogenous over-expression of TRIM2 significantly inhibited the proliferation, migration and invasion of RCC cells, suggesting that the positive regulation of TRIM2 might be a novel therapeutic target for ccRCC [30]. Kempkensteffen et al. revealed that XAF1 significantly increased the relative risk of tumor recurrence and tumor-related death [31]. Another four genes, GBP1, GBP5, LY6E and OAS2, have also been reported to be associated with a variety of other tumor progression [32–36].

Based on the functional annotation and pathway enrichment analysis of TRIM2 co-expressed genes, the mechanism of TRIM2 regulating RCC development was intuitively outlined. The results suggest that TRIM2 can positively activate ubiquitin mediated protein hydrolysis and tight junction pathway. In addition, TRIM2 may inhibit cytokine−cytokine receptor interaction, cell adhesion molecules (CAMs), Th17 cell differentiation, as well as Th1 and Th2 cell differentiation signaling pathway. Some ubiquitinases promote tumorigenesis by controlling the ubiquitination level of the tumor suppressor or carcinogenic substrate [37–40]. They catalyzed the ubiquitination of molecules which were closely related to the regulation of cell fate, growth, differentiation and apoptosis. Therefore, the abnormal expression, mutation and unregulated activity of these enzymes are related to the development of cancer and chemotherapy resistance. Many molecules associated with cell adhesion, such as integrin family, determine the colonization of metastasis and promote the survival of circulating tumor cells independent of anchoring [41]. Integrin mediated sensing, hardening and remodeling of tumor stroma serve as key steps to support tumor invasion, dryness and drug resistance. With regards to T-helper 17 (Th17), current research has been focused on basic biology, pedigree development, inflammation promotion and autoimmune correlation [42]. The tumor promoting mechanism of Th17 cells is believed to be related to the production of angiogenesis, immunosuppressive cytokines and chemokines in tumor micro-environment (TME), which can induce tumor growth and metastasis [43, 44]. IL-23, a cytokine, may selectively expand CD4 + T cell groups which express IL-17 and participate in Th17 differentiation pathway [32]. TRIM2 may inhibit the progression and metastasis of kidney cancer through ubiquitinated modification of cell adhesion molecules and Th17 differentiation key proteins.

However, there remain several limitations in our research. First of all, our ceRNA network is based on bioinformatics analysis; the mRNA regulatory network of circRNA / miRNA needs to be verified both in vitro and in vivo. Secondly, due to the deficient relevant prognostic data, the prognostic value of circRNAs in ccRCC patients cannot be evaluated. Thirdly, our sample size is relatively small.

A ceRNA network is identified which describes the possible mechanism of ccRCC. Our study provides a mechanistic insight into the unknown regulatory network of ceRNAs in ccRCC. Multivariate analysis showed that TRIM2 was an independent prognostic factor, suggesting that the hsa_circ_0002286 / has-mir-222-5p / TRIM2 axis might play a inhibitory role in the development and metastasis of ccRCC. Hence, TRIM2 may serve as a potential target to inhibit the progression and metastasis of renal cell carcinoma. The specific mechanism needs further elucidation.

## MATERIALS AND METHODS

### Data acquisition

The circRNA expression profiles for human samples derived from patients with kidney cancer were obtained from GEO database (http://www.ncbi.nlm.nih.gov/geo). We selected the GSE100186 and GSE137836 circRNA expression profiles, which were based on the same platform: GPL21825 074301 Arraystar Human CircRNA microarray V2. The GSE100186 dataset included four clear cell renal cell carcinoma tissues and four adjacent normal tissues, and the GSE137836 dataset included three metastatic renal cell carcinoma tissues and three primary tumor tissues. Additionally, the transcriptome data of ccRCC used in this study was derived from TCGA. We downloaded the transcriptome data of ccRCCs from the TCGA database through the R package, including 72 cases of adjacent normal tissues and 537 cancer cases. Furthermore, relevant clinical information including age, gender, stage, tumor/ Lymph node/ metastasis staging, and survival status were obtained duration as well (Table 4). "Limma" package in R software was utilized to correct the transcriptome data we have downloaded. We obtained the drug sensitivity data of ccRCC cell line from the GDSC database (https://www.cancerxgene.org/). The results of immunohistochemical expression of the hub genes in ccRCC and adjacent tissues were obtained by HPA database.

**Table 4:** Clinicopathological characteristics of patient samples in TCGA KIRC.

**Characteristics**	**Number of cases (%)**
**Age (y)**	
≥60	271(50.5)
<60	266(49.5)
**Gender**	
Male	346(64.4)
Female	191(35.6)
**Pathologic grade**	
G1	14(2.6)
G2	230(42.8)
G3	207(38.6)
G4	78(14.5)
GX/Unknow	8(1.5)
**Clinical stage**	
I	269(50.1)
II	57(10.6)
III	125(23.3)
IV	83(15.5)
Unknow	3(0.5)
**T classification**	
T1	275(51.2)
T2	69(12.8)
T3	182(33.9)
T4	11(2.0)
**N classification**	
N0	240(44.7)
N1	17(3.2)
NX/Unknow	280(52.1)
**Metastasis**	
No	426(79.3)
Yes	79(14.7)
Unknow	32(6.0)
**Vital states (at follow-up)**	
Alive	367(68.3)
Dead	170(31.7)

### Prediction of MREs

The online tool-CSCD (http://gb.whu.edu.cn/CSCD/) was used to predict miRNA binding sites in the selected DECs. Moreover, we crossed the predicted target miRNAs with TCGA KIRC DEmiRs as potential target miRNAs for circRNAs.

### Identification of differentially expression RNAs and enrichment analysis

Differential expression of the circRNAs in the two datasets was analyzed using GEO2R online analysis tool (http://www.ncbi.nlm.nih.gov/geo/geo2r). The criteria for significance were |log 2 (fold change [FC])|> 1 and P-value<0.05. Then, we integrated and ranked all of DECs from two circRNA chips with R package RobustRankAggreg. The HTSeq-Counts of miRNAs and mRNAs in TCGA-KIRC were analyzed to identify DEmiRs and DEmRs by utilizing the edger package. To detect more significant genes, values of |log 2 (fold change[FC])|>1 and P-value<0.05 were set as cut-off criteria. Venn diagrams were constructed to determine intersections via VENNY 2.1 software (http://bioinfogp.cnb.csic.es/tools/index.html).

### Prediction of miRNAs target mRNAs

The miRNA-mRNA interactions were respectively predicted by three online websites (miRDB (http://www.mirdb.org/), TargetScan (http://www.targetscan.org/), and DIANA (http://diana.imis.athena-innovation.gr/) databases). Target genes predicted consistently by the three databases were selected. Furthermore, we crossed the predicted target genes with TCGA KIRC DEmRs as potential target genes for miRNAs.

### GO and KEGG pathways of mRNAs in the network

GO analysis was used for functional analysis of the genes and consisted of BP, CC and MF. KEGG pathway analysis revealed signaling pathway information for the genes. All DEGs in the network were analyzed with the DAVID online database (https://david.ncifcrf.gov/) in the GO and KEGG pathway methods. The results were download and visualized with the ggplot2 R package.

### Construction of circRNA-miRNA-mRNA network

The network consisted of potential target genes to the miRNAs, potential target miRNAs to the DECs and corresponding DECs. Cytoscape software (version 3.7.2) was utilized to visualize the circRNA-miRNA-mRNA network.

### Construction of PPI network and identification of hub-genes

Using the Search Tool for the Retrieval of the Interacting Genes (STRING) (v11.0) (https://string-db.org/), a PPI network was built based on DEGs in the circRNA-miRNA-mRNA network. Visualization of the network was accomplished using Cytoscape. The Cytoscape plugin “MCODE” was used to identify significant module and hub genes, with cut-off criteria of degree≥2 and k-core≥2.

### Survival analysis and expression comparison of hub-genes

Clinical data for TCGA-KIRC including survival time, survival status, and TNM stage were also downloaded from the TCGA database (samples with missing information were excluded). Survival R package was applied in survival analyses for hub-genes. For the overall survival rates, the Log-rank test was used to detect significant differences. The results were visualized by Kaplan-Meier survival curves, and P-value<0.05 was considered as statistically significant.

### Gene set enrichment analysis

Gene enrichment analysis (GSEA) (version 3.0, the broad institute of MIT and Harvard, http://software.broadinstitute.org/gsea/downloads.jsp) was conducted to study the biological characteristics of hub genes in ccRCC. In details, the “collapse data set to gene symbols” was set to false, the number of marks was set to 1000, the “permutation type” was set to phenotype, the “enrichment statistic” was set to weighted, and the Signal2Noise metric was used for ranking genes. High expression group was used as experimental group and low expression group was used as reference group. “c2.cp.kegg.v7.0.symbols.gmt” gene sets database was used for enrichment analysis. Gene set size >500 and <15, FDR <0.25, and nominal P-value <0.05 were regarded as the cut-off criteria.

### Cell culture and regents

786-O, Caki-1 and 293T cell lines were obtained from the University of Colorado Cancer Center Cell Bank. 786-O&293T cell and Caki-1 cell were cultured in RPMI 1640 medium and 5A medium mixed with 10% FBS (Invitrogen, Carlsbad, CA, USA) at a circumstances of 37° C and 5% CO_2_ atmosphere, respectively. All experiments were performed with mycoplasma-free cells.

### RNA extraction, reverse transcription and quantitative PCR (RT-qPCR)

A total of 15 paired fresh-frozen ccRCC tissues and normal tissues were obtained from patients diagnosed with ccRCC at the The First Affiliated Hospital of Nanjing Medical University. Total cellular RNA from tissues and cells was collected with Trizol Regent (Invitrogen). cDNA was reversed from total RNA with PrimeScript™ RT reagent kit (Takara Bio, Inc., Otsu, Japan). Real-time quantitative PCR was utilized to assess the circ_RNAs expression, which was carried out in triplicate by a SYBR Premix Ex Taq™ kit (Takara Bio) and ABI 7900HT Real-Time PCR system (Applied Biosystems Life Technologies, Foster City, CA, USA). The primers applied are displayed in Table 5. The final results were analyzed through the comparative cycle threshold values (2-ΔΔCt).

**Table 5:** Primers for RT-qPCR.

**Gene**	**Primer**	**Sequence (5′-3′)**
hsa_circ_0002286	Forward	TTGGCACGCTACTTTTACGC
	Reverse	GGGTACTCGCATCACACTCA
hsa_circ_0000512	Forward	CTCCTTTGCCGGAGCTTG
	Reverse	GGTCCACGGCATCTCCTG
hsa_circ_0003596	Forward	CCCTGACAAGAAGTCCGAAGG
	Reverse	TCCTGGATGCCTGGATTGG
hsa_circ_0001968	Forward	TGATGTCTGGAGCACAGAGG
	Reverse	TCAGAGCCACCATCATCAAA
hsa_circ_0037866	Forward	GCAAGGATTGACGTGTCTCA
	Reverse	CAGGTGGCTTTGCTGGTATT
hsa_circ_0008259	Forward	AAGAAGCCCAGCTTTTCCAT
	Reverse	TCACACAGCAGAACACCATTT
hsa_circ_0058792	Forward	GCTCTCCAACGACCTGAAAC
	Reverse	GACATATGTGCCCGTCAGGT
hsa_circ_0035436	Forward	GAAGAGGTTTCCAGCCATGA
	Reverse	CTTGCTCCTCTTTGGCAATC
hsa-mir-222-5p	Stem-loop	GTCGTATCCAGTGCAGGGTCCGAG GTATTCGCACTGGATACGACAGGATC
	Forward	CAGCACTCAGTAGCCAGT
TRIM2	Forward	CCACTCTTCAGCACATTCC
	Reverse	GCCACCACCGTCATTC
Actin	Forward	AGCGAGCATCCCCCAAAGTT
	Reverse	GGGCACGAAGGCTCATCATT

### Statistical analysis

All analyses were performed using R 3.6.1. All statistical tests were two-sided, and P value <0.05 was considered statistically significant. Continuous variables that conformed to the normal distribution were compared with the use of independent t test for comparison between groups, while continuous variables with skewed distribution were compared with the Mann-Whitney U test. The relationship between hub genes and overall survival was analyzed through the Kaplan-Meier (KM) curve which was evaluated by log-rank test. The univariate regression model was used to analyze the effects of individual variables on survival, and the multivariate cox regression model was used to confirm independent impact factors associated with survival.

### Ethics approval

All the patients provided written informed consent, and the protocol was approved by ethical committee of The First Affiliated Hospital of Nanjing Medical University.

## Supplementary Material

Supplementary Figures
