# Supplementary material for: Construction of circRNA-based ceRNA network to reveal the role of circRNAs in the progression and prognosis of metastatic clear cell renal cell carcinoma
Source: Aging (Albany NY). 2020 Nov 20;12(23):24184–207. doi: 10.18632/aging.104107 (PMC7762515; doi:10.18632/aging.104107)
Supplement: Supplementary Figures [file aging-12-104107-s001.pdf]

SUPPLEMENTARY FIGURES

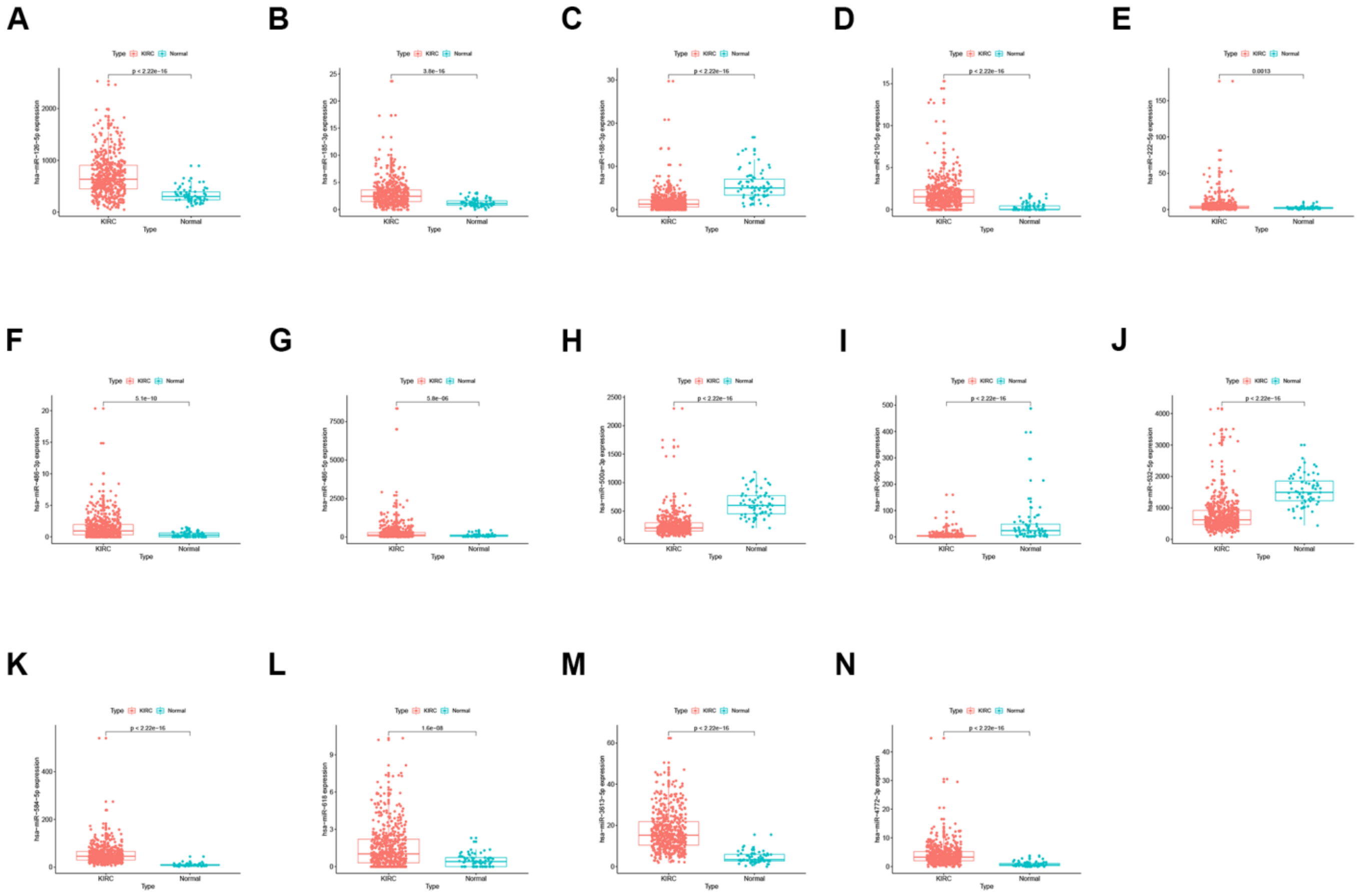

Supplementary Figure 1. The expression of 14 miRNAs in ccRCC tissues and normal kidney tissues. (**A**) hsa−miR−126−5p. (**B**) hsa−miR−185−3p. (**C**) hsa−miR−188−3p. (**D**) hsa−miR−210−5p. (**E**) hsa−miR−222−5p. (**F**) hsa−miR−486−3p. (**G**) hsa−miR−486−5p. (**H**) hsa−miR−500a−3p. (**I**) hsa−miR−509−3p. (**J**) hsa−miR−532−5p. (**K**) hsa−miR−584−5p. (**L**) hsa−miR−618. (**M**) hsa−miR−3613−5p. (**N**) hsa−miR−4772−3p. KIRC: kidney renal clear cell carcinoma.

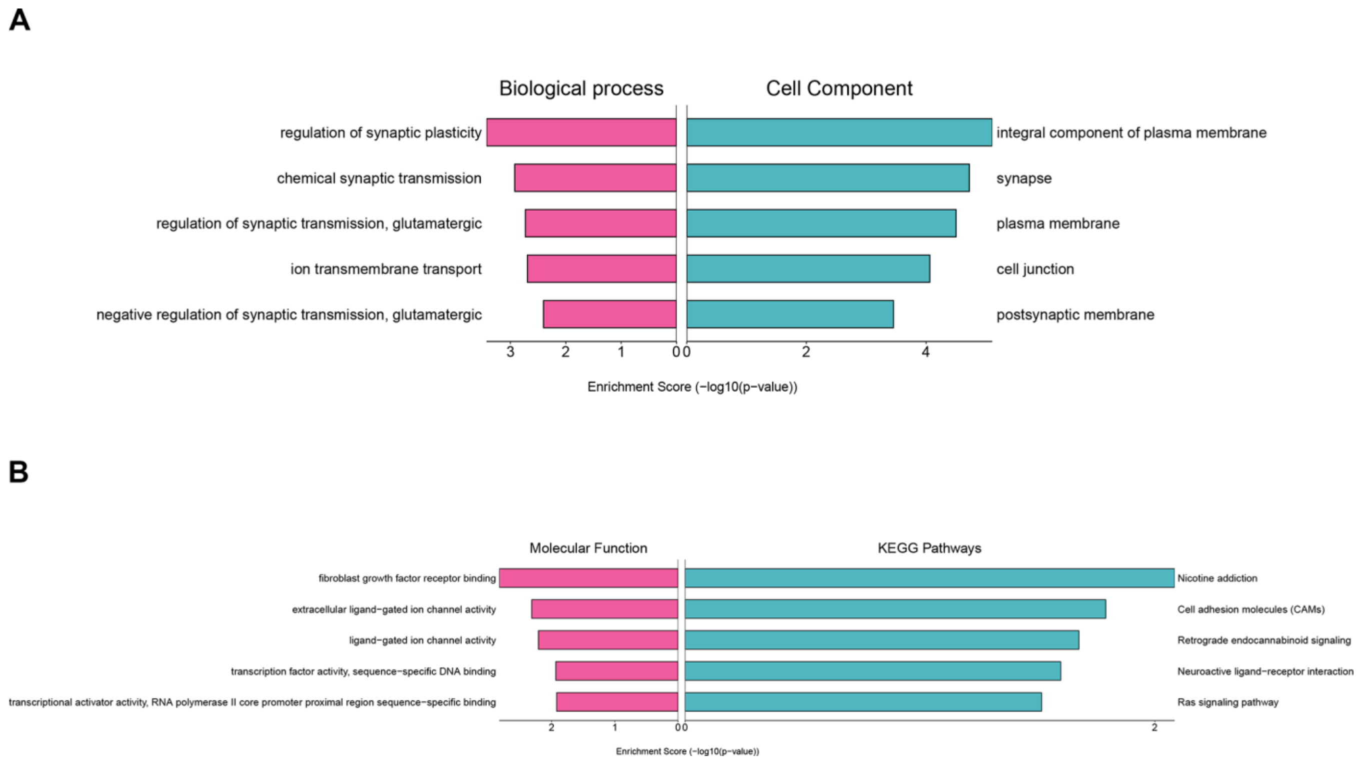

Supplementary Figure 2. Enrichment of the GO terms and KEGG pathways of 201 differentially expressed target mRNAs. (**A**). Biological process and Cell Components. (**B**). Molecular Function and KEGG Pathways. GO: Gene Ontology; KEGG: Kyoto Encyclopedia of Genes and Genomes.

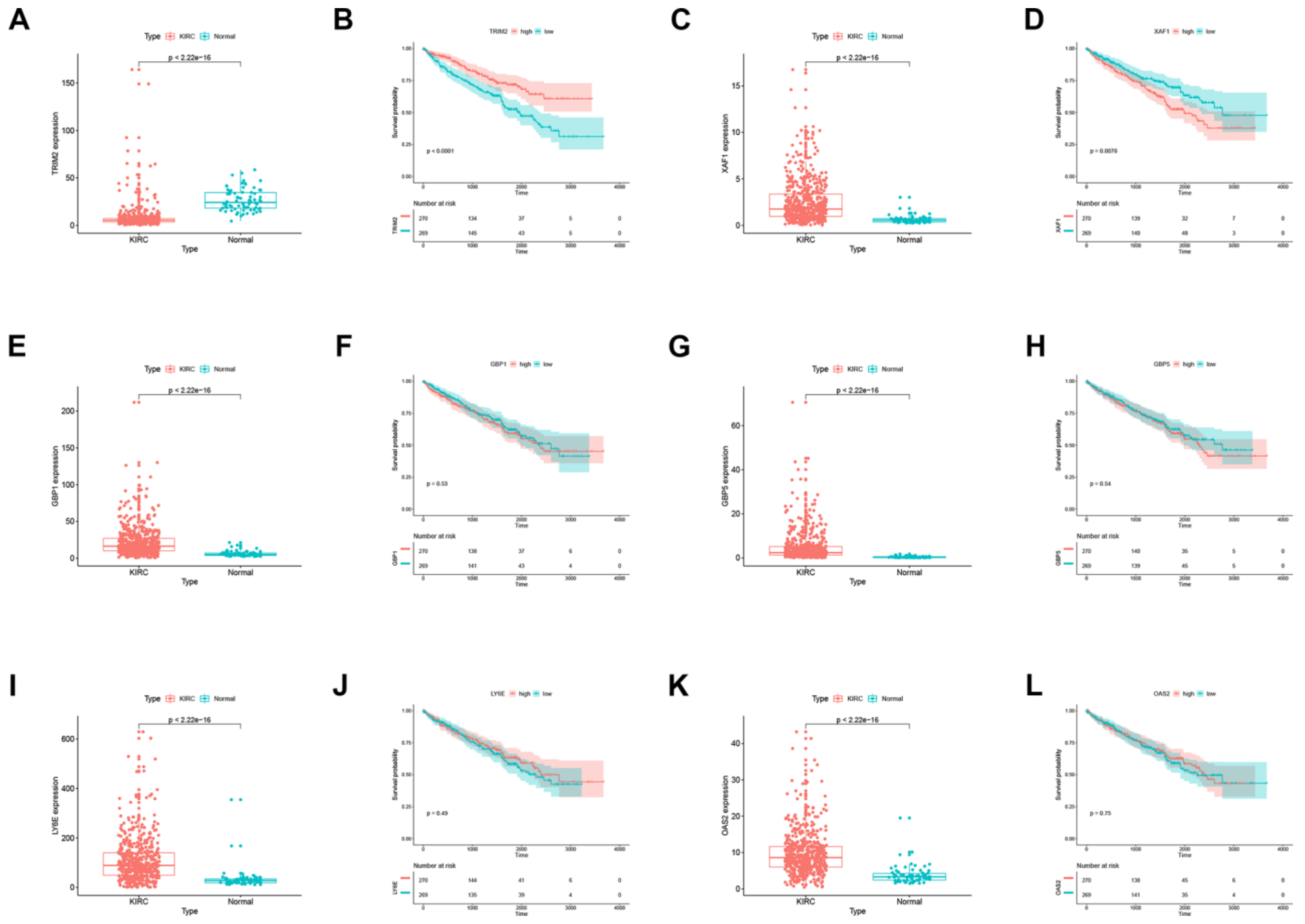

Supplementary Figure 3. The expression level and survival analysis of six hub genes in ccRCC tissues and normal kidney tissues. (**A**) The expression level of TRIM2 between KIRC and normal samples. (**B**) The survival analysis of TRIM2 in TCGA KIRC patients. (**C**) The expression level of XAF1 between KIRC and normal samples. (**D**) The survival analysis of XAF1 in TCGA KIRC patients. (**E**) The expression level of GBP1 between KIRC and normal samples. (**F**) The survival analysis of GBP1 in TCGA KIRC patients. (**G**) The expression level of GBP5 between KIRC and normal samples. (**H**) The survival analysis of GBP5 in TCGA KIRC patients. (**I**) The expression level of LY6E between KIRC and normal samples. (**J**) The survival analysis of LY6E in TCGA KIRC patients. (**K**) The expression level of OAS2 between KIRC and normal samples. (**L**) The survival analysis of OAS2 in TCGA KIRC patients. TCGA: the cancer genome atlas; KIRC: kidney renal clear cell carcinoma.

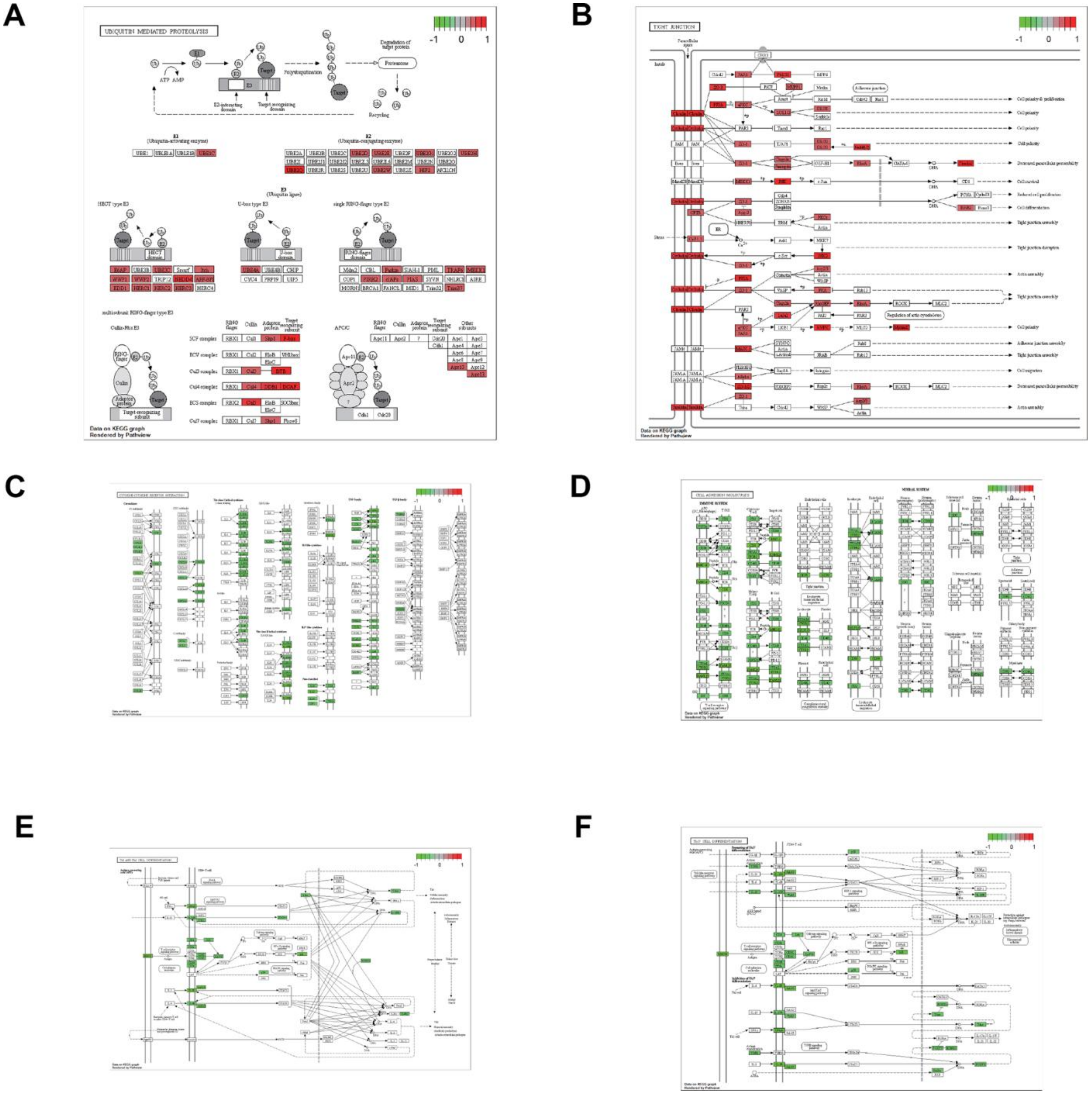

Supplementary Figure 4. Potential KEGG pathways TRIM2 was involved in. (**A**) UBIQUITIN MEDIATED PROTEOLYSIS. (**B**) TIGHT JUNCTION. (**C**) CYTOKINE-CYTOKINE RECEPTORINTERACTION. (**D**) CELL ADHESION MOLECULES. (**E**) Th1 AND Th2 DIFFERENTIATION. (**F**) Th17 CELL DIFFERENTIATION. KEGG: Kyoto Encyclopedia of Genes and Genomes.

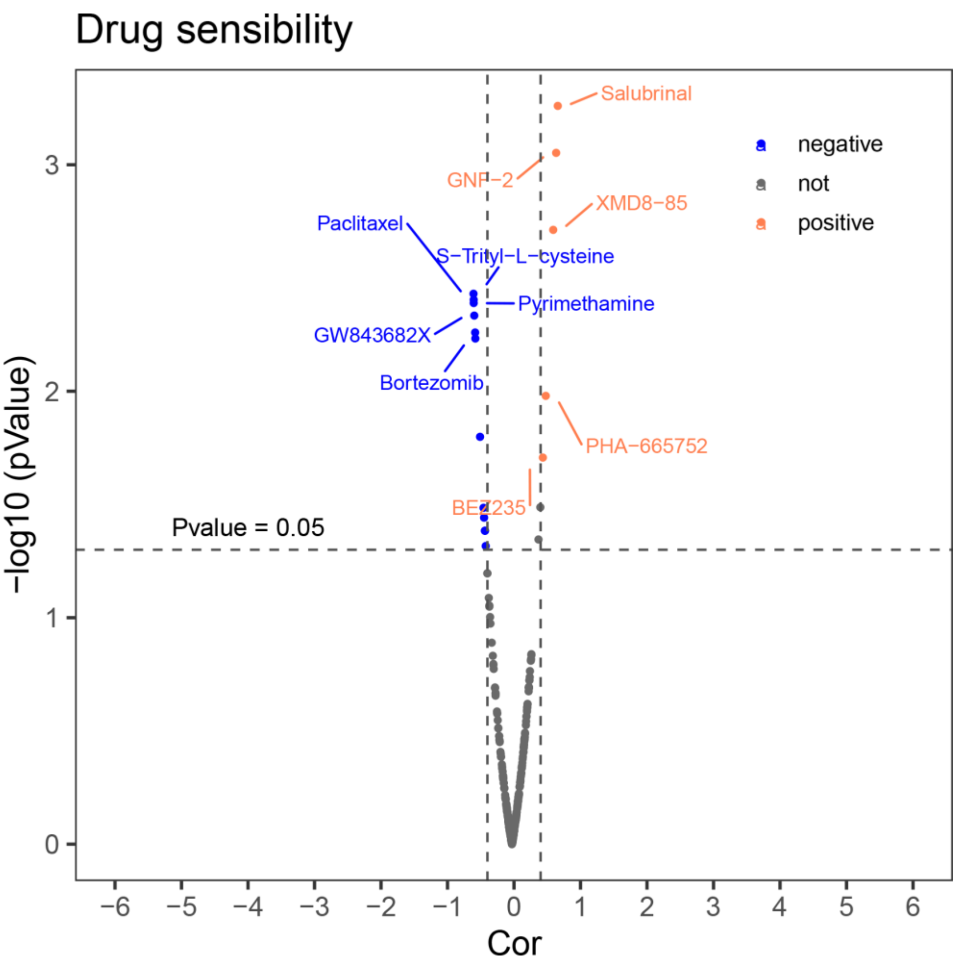

Supplementary Figure 5. Correlation between the expression status of TRIM2 and drug sensitivity of ccRCC cell lines.
